# Preparation of Microcapsules Containing β-Carotene with Thermo Sensitive Curdlan by Utilizing Reverse Dispersion

**DOI:** 10.3390/pharmaceutics5040609

**Published:** 2013-11-21

**Authors:** Yoshinari Taguchi, Fumiyasu Ono, Masato Tanaka

**Affiliations:** 1 Department of Chemistry and Chemical Engineering, Niigata University, 8050, Ikarashi 2-nocho, Niigata-shi, Niigata 950-2181, Japan; E-Mail: puchi@eng.niigata-u.ac.jp; 2 Collaborative Research Division Art, Science and Technology Center for Cooperative Research, Kyushu University, 4-1, Kyudaishinmachi, Nishi-ku, Fukuoka 819-0388, Japan; E-Mail: ono@astec.kyushu-u.ac.jp

**Keywords:** curdlan microcapsule, reverse dispersion, thermogelation, β-carotene, liquid liquid dispersion

## Abstract

We have tried to microencapsulate β-carotene with curdlan of a thermogelation type polysaccharide. Microcapsules were prepared by utilizing reverse dispersion, in which salada oil was the continuous phase (O’) and the curdlan water slurry (W) was the dispersed phase. β-carotene (O) as a core material was broken into fine oil droplets in the dispersed phase to form the (O/W) dispersion. The (O/W) dispersion was poured in the continuous phase (O’) and stirred to form the (O/W)/O’ dispersion at room temperature and then, temperature of the dispersion was raised to 80 °C to prepare curdlan-microcapusles containing β-carotene. In this microencapsulation process, the concentrations of curdlan and oil soluble surfactant and the impeller speed to form the (O/W)/O’ dispersion were mainly changed stepwise. We were able to prepare microcapsules by the microencapsulation method adopted here. The content of core material was increased with the curdlan concentration and decreased with the impeller speed and the oil soluble surfactant concentration. With the curdlan concentration, the drying rate of microcapsules was decreased and the retention ability for water was increased due to the stable preservation of β-carotene.

## 1. Introduction

Microcapsules have been applied in many fields such as cosmetics, information-recording materials, paintings, catalyst, food industry, medicine, agriculture, adhesives and so on [1,2,3,4,5,6]. The main purposes of microencapsulation are to protect the core material from environment, to enable controlled release the core material, to modify the surface of core material, to mask the taste of core material, and so on. It is well known that these functions of microcapsules are strongly dependent on the structure of microcapsules and the physical properties of shell materials such as gelation activity, reactivity, responsibility to various stimuli, organic solvent proof, acid and base proof, permeability for *oxygen* and water vapor and so on [7,8,9]. The type of structure of microcapsule that is constructed is dependent on the preparation method and the physical properties of core and shell materials. Accordingly, in order to give the microcapsules the desired functions, it is necessary to develop the preparation method which is suitable to the core and shell materials used.

Many kinds of polysaccharides have been used as the microcapsule shell materials because of their stimuli responsibility. A few polysaccharides with thermal gelation among them are used to microencapsulate the oily core materials by using the reverse emulsion system. Namely, the polysaccharide aqueous solution, in which oily core material is dispersed in the fine oil droplets, is stirred in the continuous oil phase to form the (O/W)/O’ dispersion. Then, the temperature of this dispersion is raised or lowered to the gelation temperature to form the microcapsules containing the oily core material. Michael *et al.* have prepared whey protein microcapsules containing bilberry anthocyanins by using the thermal gelation of whey protein aqueous solution and investigated the effect of preparation conditions on the diameter of microcapsules and the content of core material [7,8]. Curdlan of a water insoluble polysaccharide has the unique property that curdlan water slurry results in reversible gelation at 60 °C and irreversible gelation above 80 °C. By applying this unique property, it may be expected to prepare microcapsules responsible to temperature or with the thermostable shell.

On the other hand, β-carotene as a model core material is known to have many physiological activities such as anti-aging effect and preventive effect for cancer, but thermostability is very low. Accordingly, if β-carotene is able to be microencapsulated by the thermostable shell material, application of β-carotene has to be extended. Furthermore, if another hydrophobic materials are able to be contained into β-carotene, the multiple functions may be given to the microcapsules.

The purposes of this study are to investigate whether or not β-carotene is able to be microencapsulated with curdlan by utilizing the reverse dispersion, to discuss how the preparation conditions affect a few characteristics of microcapsules and to develop the preparation method by using the designated materials.

## 2. Materials and Methods

### 2.1. Materials

**Figure 1:**
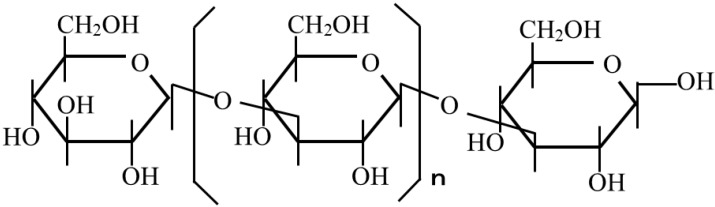
Molecular structure of Curdlan.

Curdlan of thermal gelation type polysaccharide, which are produced by Zymogens such as *Agrobacterium* and *Alcaligenes*, was used as the shell material (Takeda Medical Supply Industries, Osaka, Japan). The molecular structure of curdlan is shown in Figure 1. The core material was β-carotene (Wako Pure Chemical Industries, Tokyo, Japan). Soybean lecithin was used as an oil soluble surfactant (Kanto Chemicals Industries, Tokyo, Japan). Salada oil was used as the continuous phase. Distilled water was used as the dispersed phase.

### 2.2. Preparation of Microcapsules

**Figure 2:**
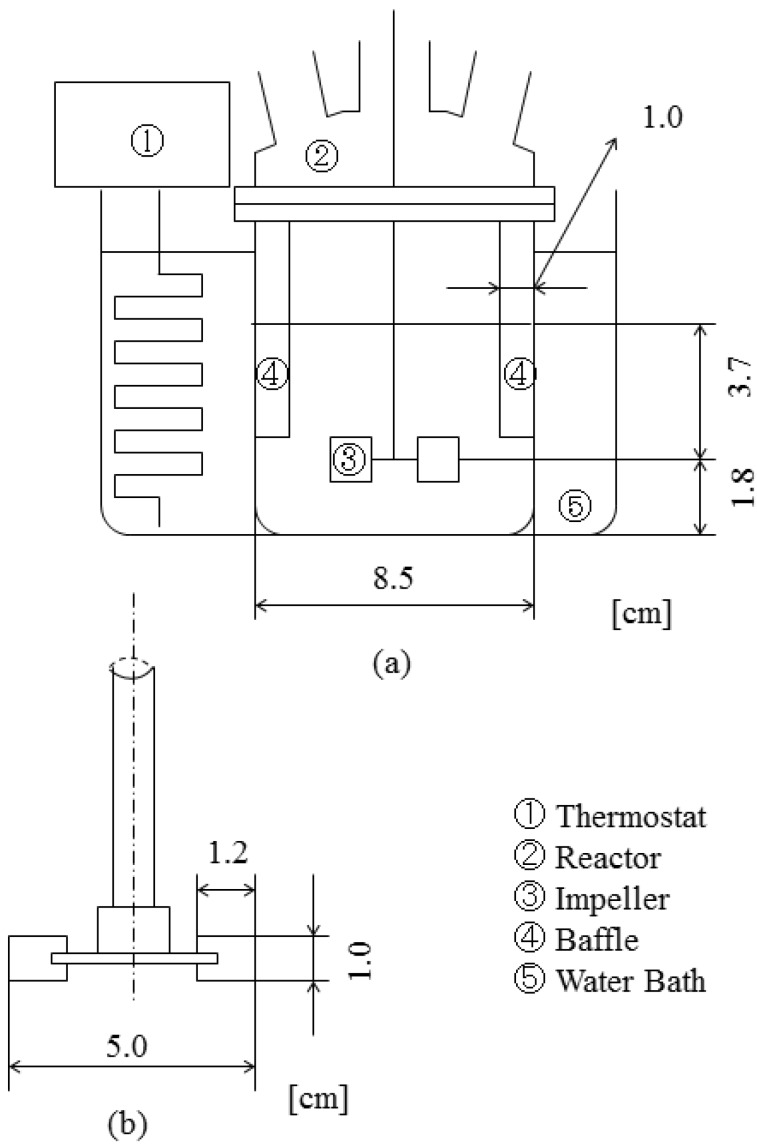
Schematic diagrams of experimental apparatus.

Figure 2 shows the schematic diagrams of experimental apparatus used in this study. A separable flask with a flat bottom was used as the reactor to prepare the microcapsules. Four baffles made of aluminum plate with width of 1 cm and length of 6 cm were installed on the inside wall of the reactor, as shown in Figure 2a. The impeller used in the microencapsulation process was a six-blade disc turbine with the diameter of 5.0 cm and width of 1.2 cm as shown in Figure 2b, which was fixed at the height of 1.8 cm from the reactor bottom. A rotor-stator type homogenizer was used to prepare the curdlan water slurry. The thermostatted water bath was used to control temperature of the dispersion.

**Figure 3:**
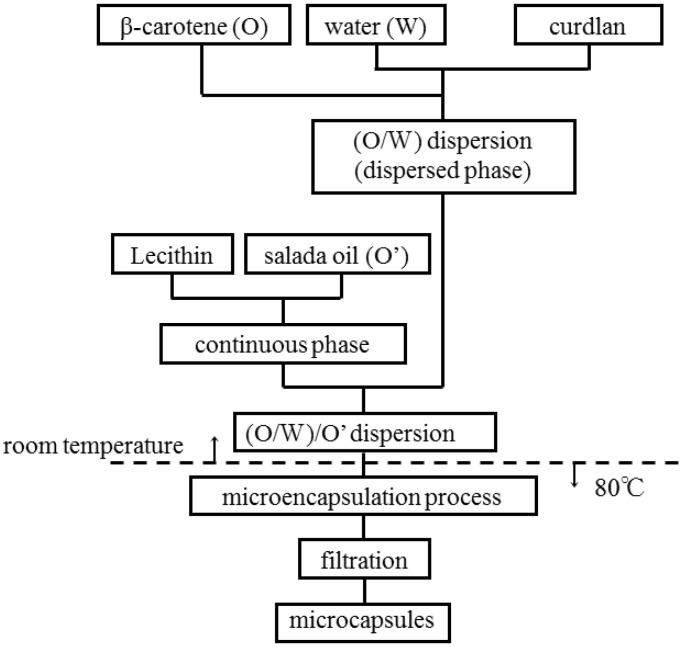
Flow chart for preparing microcapsules.

Figure 3 shows the flow chart for preparing the microcapsules by utilizing reverse dispersion. Namely, β-carotene of a given weight was dispersed to form the (O/W) dispersion under stirring in the water phase, where curdlan of a given amount was suspended. The (O/W) dispersion thus formed was used as the dispersed phase. On the other hand, salada oil (O’) dissolving lecithin of a given concentration was used as the continuous phase, into which the dispersed phase was poured to form the (O/W)/O’ dispersion. After this operation, the temperature of the dispersion was raised from room temperature to 80 °C and stirred for 30 min to prepare the microcapsules with irreversible gelation of curdlan.

In this fundamental microencapsulation process, the impeller speed to form the (O/W)/O’ dispersion and the concentrations of curdlan and lecithin were changed stepwise. Experimental conditions are shown in Table 1. Here, the impeller speed is defined as the number of rotation of impeller per minute.

**Table 1:** Experimental conditions for preparation of microcapsules.

Items	Conditions
Continuous phase (salada oil)	300 cc
Oil soluble surfactant (soybean lecithin)	*C*_L_ = 0–1 [wt%-oil]
Core material (β-carotene)	0.1 g
Shell material (curdlan)	*C*_C_ = 2.0–7.0 [wt%-H_2_O]
Hold up of dispersed phase	0.1
Impeller speed at (O/W) dispersion formation	3000 rpm
Stirring time for (O/W) dispersion formation	15 min
Impeller speed at (O/W)/O’ dispersion formation	200–600 rpm
Time for microencapsulation process	30 min
Temperature	80 °C

### 2.3. Characterization

Microcapsules prepared were observed by optical microscope (BH-2; OLYMPUS Corp, Tokyo, Japan), and microphotographs were taken. From these microphotographs, the mean diameters were estimated by measuring the diameter of 100 microcapsules and averaging these values. Here, the mean diameter is the mean Sauter diameter.

Viscosities of curdlan slurry and salada oil dissolving lecithin were measured by the plate vibrating viscometer (VM-1A, Yamaichi Electric Ind. Co., Ltd., Tokyo, Japan). Interfacial tension between the continuous phase and the dispersed phase was measured by Auto Tension Meter (CBVP-A3, Shimazu Seisakusho Ind. Co., Ltd., Kyoto, Japan).

Microcapsules of a given weight were broken in the continuous phase (O’) by mechanical force with the medical spoon.

Thus, the amount of β-carotene leaked to the continuous phase was measured by UV spectrometer (UV-160A, Shimazu Seisakusho, Ind. Co., Ltd., Kyoto, Japan). For this measurement, the correlation curve between the β-carotene concentration in the continuous phase and the absorption degree of UV was drawn beforehand.

From these results, the content (*F*_C_) of core material is defined as follows.

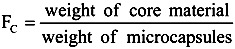
(1)


Retention ability (*R*_C_) for the core material after drying microcapsules was measured as follows. Microcapsules of a given weight were added into 20 cm^3^ of water under stirring by magnetic stirrer. After 1 h, the water phase was mixed with salada oil for 30 min to extract β-carotene. Then, the amount of β-carotene leaked was measured by the same method as stated above. Thus, retention ability for the core material is defined as follows.

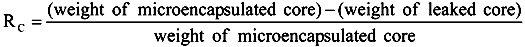
(2)


The transient weight of microcapsules of a given weight was measured at the constant time interval in the constant temperature and humidity room (*T* = 50 °C, 30%).

The weight fraction (*Y*) is defined as follows.

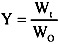
(3)


The drying rate is defined as follows.

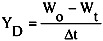
(4)


Here, *W*_t_ and *W*_o_ are the weight of microcapsules at any time and before drying, respectively and ∆*t* is the time span measuring the weight of microcapsules.

## 3. Results and Discussion

### 3.1. Observation of Microcapsules

**Figure 4:**
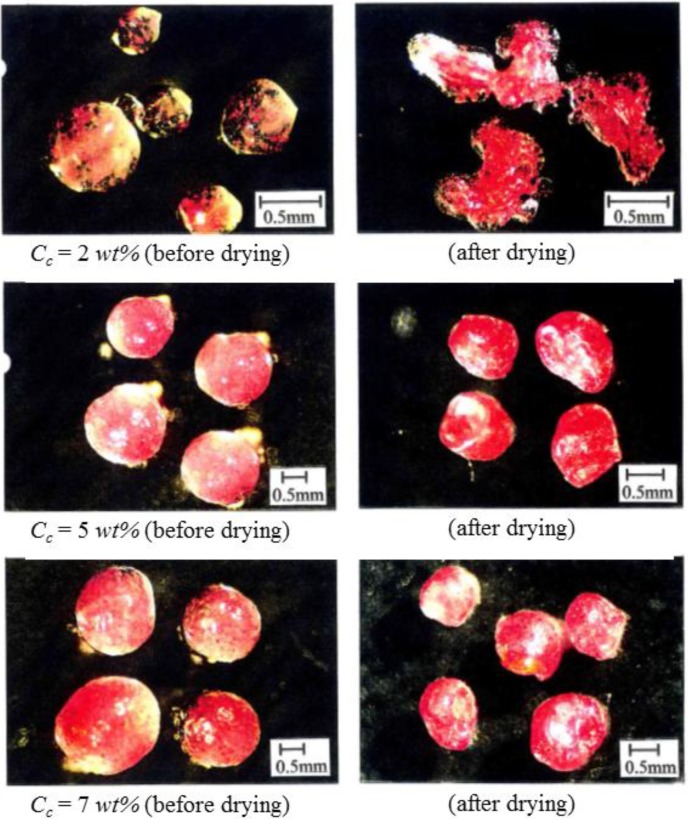
Observation of microcapsules.

Figure 4 shows the microphotographs of microcapsules before and after drying which were prepared at the various concentrations (*C*_C_) of curdlan. All microcapsules before drying were found to be almost spherical. However, the microcapsules prepared at *C*_C_ = 2 wt% are broken after drying because of weak shells due to the lower curdlan concentration.

From these results, it is found that the matrix type microcapsules are able to be prepared by the preparation method presented in this study, but there exists a critical curdlan concentration required to prepare the sound microcapsules.

### 3.2. Dependence of Mean Diameter on Impeller Speed

**Figure 5:**
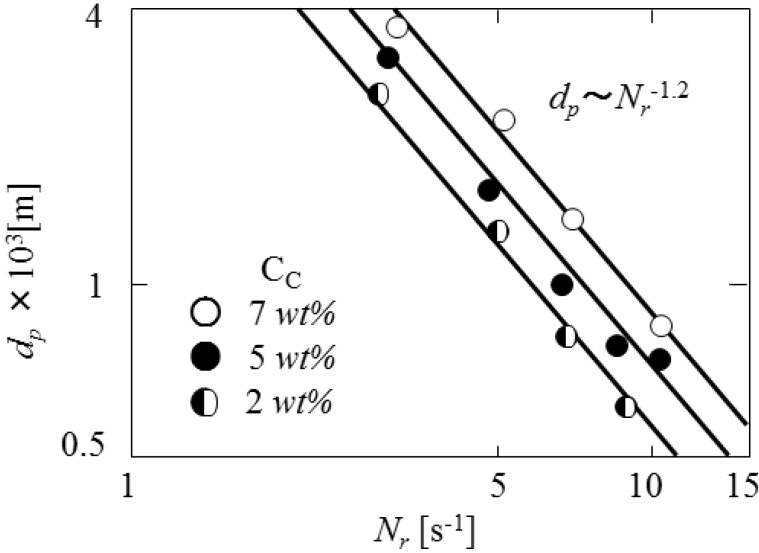
Dependence of mean diameter on impeller speed.

Figure 5 shows the dependence of the mean diameters of microcapsules on the impeller speed (*N*_r_) to form the (O/W)/O’ dispersion. The mean diameters are found to be proportional to *N*_r_^−1.2^. This dependence is in accord with that in the liquid dispersion under turbulent conditions. The diameters of (O/W) droplets in the continuous oil phase are determined by breakup dominant [10,11,12].

### 3.3. Dependence of Mean Diameter on Concentrations of Curdlan and Oil Soluble Surfactant

**Figure 6:**
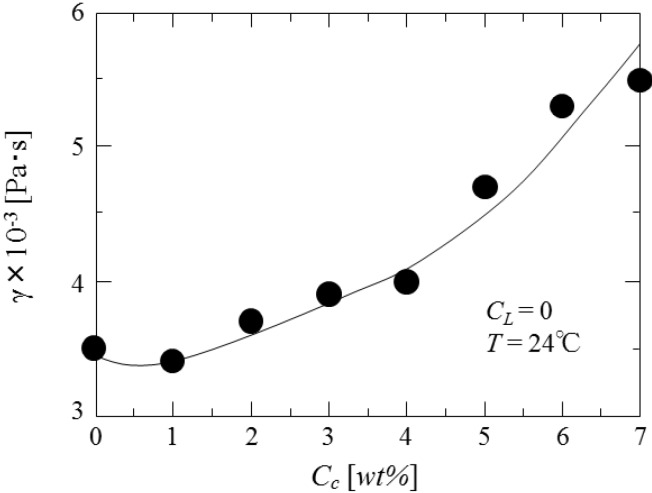
Dependence of interfacial tension on curdlan concentration.

**Figure 7:**
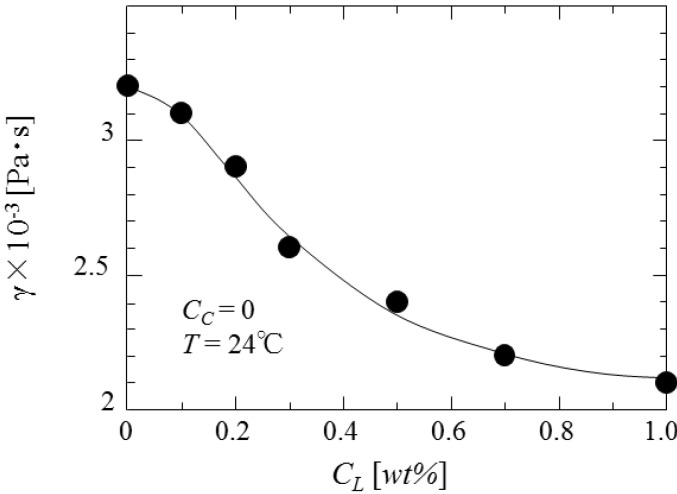
Dependence of interfacial tension on surfactant concentration.

As the diameters of droplets in the liquid dispersion are strongly dependent on the physical properties such as interfacial tension, viscosity and density of liquids concerned, first the dependence of interfacial tension on the concentrations of curdlan and oil soluble surfactant is investigated. The results are shown in Figure 6 and Figure 7, respectively. Interfacial tension is found to increase with the curdlan concentration (*C*_C_) and to decrease with the surfactant concentration (*C*_L_).

**Figure 8:**
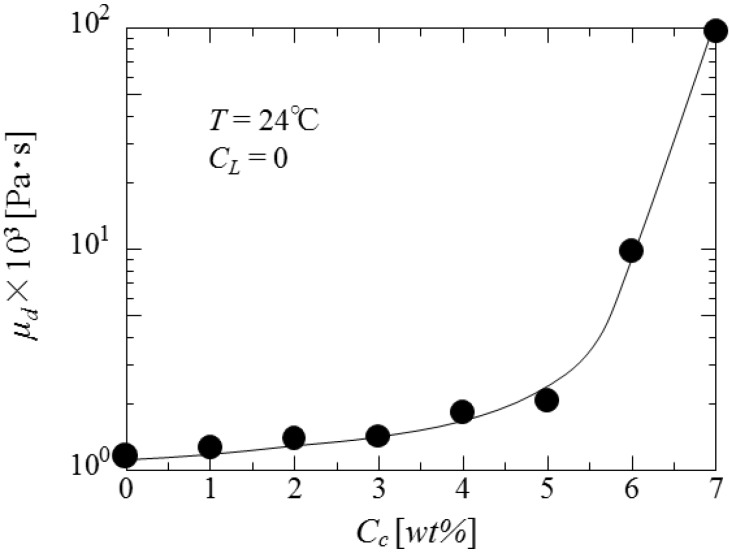
Dependence of viscosity of dispersed phase on curdlan concentration.

Figure 8 shows the dependence of viscosity of dispersed phase on the curdlan concentration. The viscosity of dispersed phase increases exponentially with the curdan concentration.

**Figure 9:**
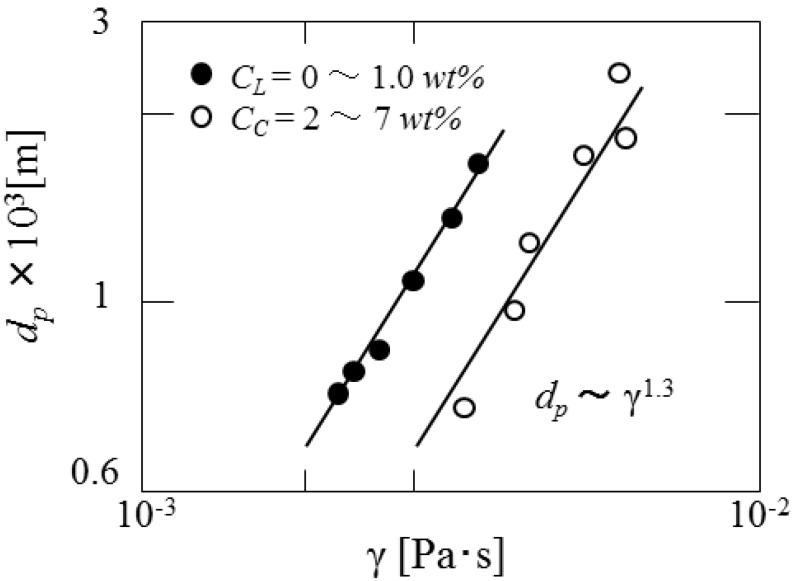
Dependence of mean diameter on interfacial tension.

Figure 9 shows the dependence of the mean diameters of microcapsules on interfacial tension changed by the concentrations of curdlan and surfactant. It is found that the mean diameters are proportional to γ^1.3^.

**Figure 10:**
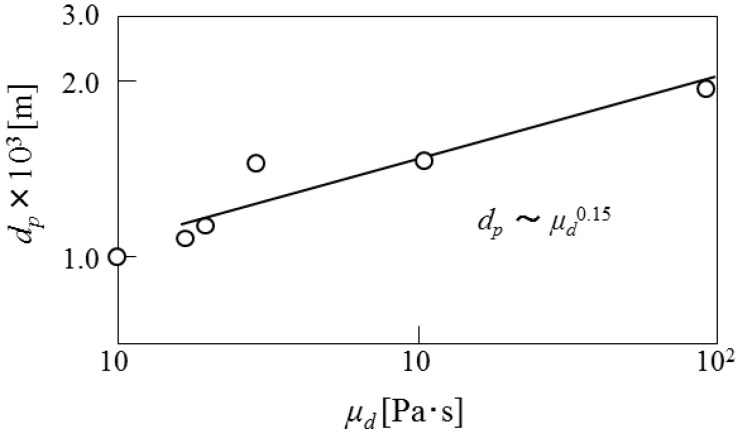
Dependence of mean diameters on viscosity of dispersed phase.

Figure 10 shows the dependence of mean diameters of microcapsules on the viscosity of dispersed phase. The mean diameters are found to be proportional to μ_d_^0.15^. From the results obtained above, the dependence of mean diameters of microcapsules on the operating conditions is derived as follows.

*d*_p_~*N*_r_^−1.2^γ^1.3^μ_d_^0.15^ (5)

The dependence of mean diameters on the impeller speed is in accordance with that in the conventional O/W dispersion, where the droplet diameters are determined under breakup dominant. However, the dependence of the mean diameter on interfacial tension is not in accordance with that in the (O/W) dispersion where the mean diameters are proportional to γ^0.6^. As the viscosity of dispersed phase also affects the droplet diameters as viscous energy against distruptive energy, it can be stated that the cohesive energy against the disruptive energy may be added to the surface energy [13,14]. As the content and the dispersion situation of β-carotene are affected by the viscosity of dispersed phase, it may be necessary to investigate how the dispersion situation of β-carotene in the curdlan water slurry is affected by the viscosity of dispersed phase in detail.

### 3.4. Dependence of Content of Core Material on Curdlan Concentration and Impeller Speed

**Figure 11:**
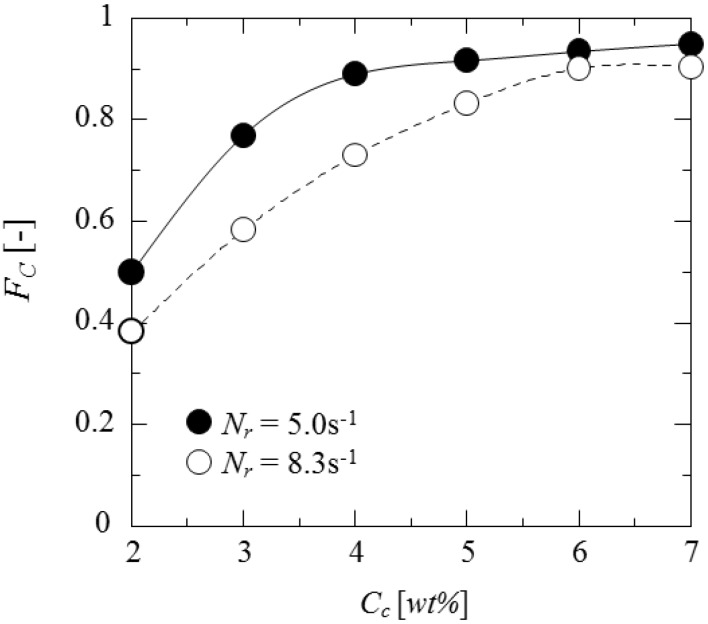
Dependence of content of core material on curdlan concentration.

Figure 11 shows the dependence of content (*F*_C_) of core material on the curdlan concentration. The content increases with the curdlan concentration and becomes constant over the 5 wt%. Although the content at *N*_r_ = 5.0 s^−1^ is larger than that at *N*_r_ = 8.3 s^−1^, this difference decreases with the curdlan concentration. This may be due to the fact that stability of oil droplets in the (O/W) dispersion and the content of core material are increased with the viscosity of curdlan water slurry (as shown in Figure 8).

**Figure 12:**
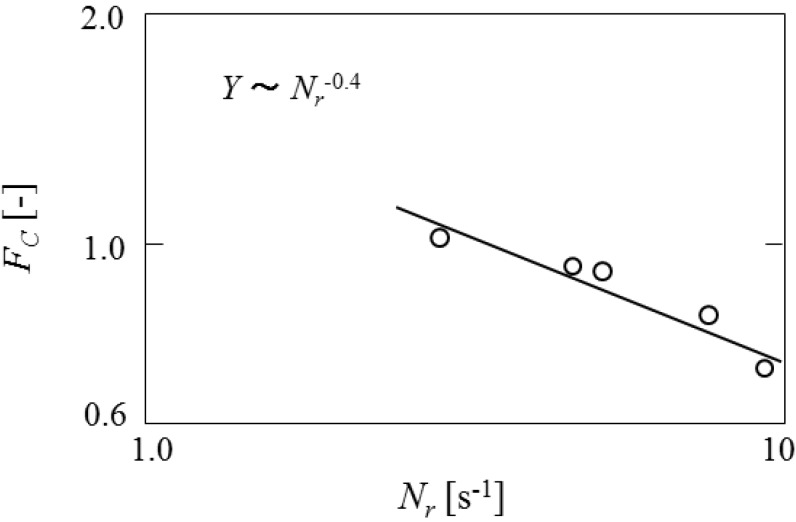
Dependence of content of core material on impeller speed.

Figure 12 shows the dependence of content of core material on the impeller speed. The content is found to decrease with *N*_r_^−0.4^. This may be due to the fact that the diameters of microcapsules are decreased with the impeller speed and the core material leaks easily from the droplets during the microencapsulation process.

### 3.5. Drying Rate and Retention Ability of Microcapsules

**Figure 13:**
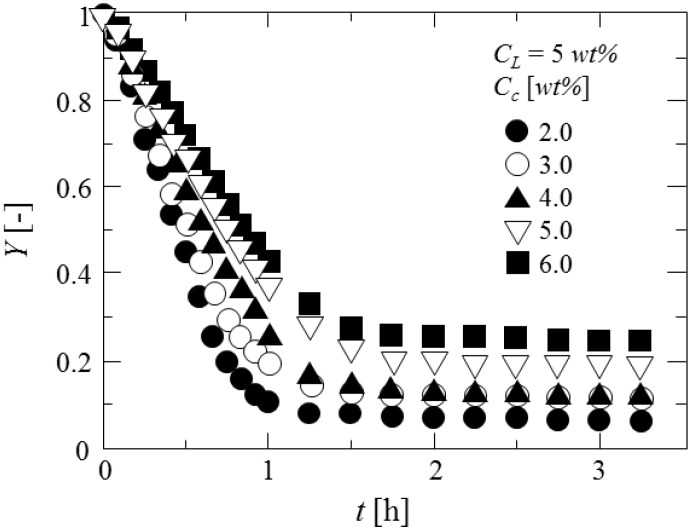
Drying feature of microcapsules.

Figure 13 shows the drying feature of microcapsules prepared at each curdlan concentration. The weight of microcapsules rapidly decreases to the equilibrium values at elapsed time of *ca.* 1 h and almost becomes constant. It is found that the lower the curdlan concentration, the higher the decreasing rate of weight of microcapsules becomes. Furthermore, the higher the curdlan concentration, the larger the equilibrium weight fraction becomes. As the shell of microcapsules becomes denser with the curdlan concentration, the release of water may be prevented.

**Figure 14:**
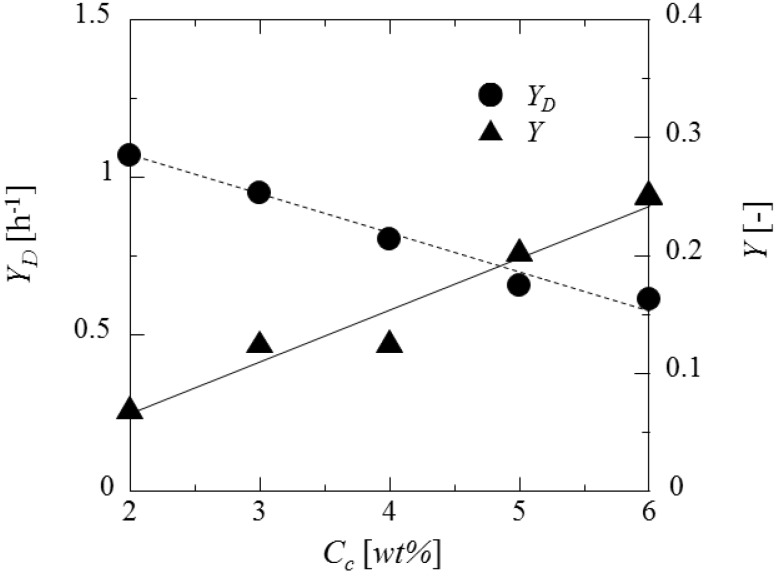
Dependence of drying rate and equilibrium weight fraction on curdlan concentration.

Figure 14 shows the dependences of the drying rate and the equilibrium weight fraction on the curdlan concentration. The drying rate is decreased with the curdlan concentration. This may be due to the fact that the microcapsule shell becomes denser with decreasing the water content. On the other hand, the equilibrium weight fraction is increased with the curdlan concentration. This may be due to the fact that retention ability of the shell for water has to increase with the curdlan concentration. For example, although the weight fraction of shell material is 6 wt% at 2 wt% of the curdlan concentration, this becomes 25 wt% at 6 wt% of the curdlan concentrations.

Table 2 shows the results of retention ability for core material. The leakage fraction of the core material is deceased with the concentrations of curdlan and oil soluble surfactant. The leakage of the core material is perfectly prevented over *C*_C_ = 5 wt% and *C*_L_ = 3 wt%. As, with the curdlan concentration, the water volume retained in the shell is decreased and the shell becomes denser, β-carotene of more hydrophobic core material may be preserved well. On the other hand, as the oil droplets of β-carotene become smaller with the concentration of oil soluble surfactant and stably disperse in the curdlan water slurry, they are stably contained in the microcapsule.

**Table 2:** Retention ability (*R*_C_).

	C_C_	2.0	3.0	4.0	5.0	6.0	7.0
C_L_							
0		0.9	0.92	0.94	1.0	1.0	1.0
0.1		0.92	0.95	0.96	1.0	1.0	1.0
0.2		0.96	0.97	0.98	1.0	1.0	1.0
0.3		1.0	1.0	1.0	1.0	1.0	1.0
0.5		1.0	1.0	1.0	1.0	1.0	1.0
0.7		1.0	1.0	1.0	1.0	1.0	1.0
1.0		1.0	1.0	1.0	1.0	1.0	1.0

## 4. Conclusions

We have tried to microencapsulate β-carotene as a core material with curdlan by utilizing reverse dispersion. The results obtained are as follows.

(1) We were able to prepare the microcapsules by the preparation method presented in this study.
(2) An experimental equation correlating the mean diameters of microcapsules and the operation conditions was derived as *d*_p_~*N*_r_^−1.2^γ^1.3^μ_d_^0.15^.
(3) The content of core material was increased with the concentrations of curdlan and oil soluble surfactant and decreased with the impeller speed.
(4) Retention ability for the core material of microcapsules was increased with the concentrations of curdlan and oil soluble surfactant.
